# Enhancing Thrust in Underwater Bio-Inspired Propulsion Fin Using Shear-Stiffening Gel

**DOI:** 10.3390/biomimetics10040198

**Published:** 2025-03-25

**Authors:** Shunichi Kobayashi, Yusuke Jonishi

**Affiliations:** 1 Institute of Textile Science and Technology, Academic Assembly, Shinshu University, Ueda 386-8567, Japan; 2 Graduate School of Science and Technology, Shinshu University, Ueda 386-8567, Japan

**Keywords:** bio-inspired propulsion, shear-stiffening gel, underwater robotics, variable stiffness fins, thrust enhancement, velocity-dependent stiffness

## Abstract

In this study, we investigated the thrust enhancement in a bio-inspired underwater propulsion fin using a shear-stiffening gel. Shear-stiffening gels exhibit velocity-dependent stiffness, i.e., they become stiffer during high-speed deformation and softer during low-speed motion, providing adaptive mechanical properties without requiring complex mechanisms. A “compound joint” incorporating a dilatant compound, a material of shear-stiffening gel, was developed and experimentally evaluated against a “rigid joint” and a flexible “urethane joint”. A speed-ratio control strategy was employed to assign faster and slower oscillations during positive and negative thrust intervals, respectively. The results demonstrated that the compound joint achieved a balance between high thrust and stable performance. Its adaptive stiffness effectively reduced deformation during high-speed oscillations, enhanced thrust while maintaining flexibility during low-speed intervals, and minimized thrust fluctuations. Compared with the rigid and urethane joints, the compound joint exhibited a superior balance between a high average thrust and low thrust variation.

## 1. Introduction

Fluid propulsion mechanisms rely on screw propellers, which are widely used in semisubmersible vessels. However, screw propellers have a significant propensity to become entangled in fishing nets and aquatic vegetation. In recent years, various devices have been proposed to mitigate the entanglement of underwater propellers [1]. Nevertheless, screw propellers pose a considerable risk of injuring aquatic organisms because of their high-speed rotating blades. Furthermore, in shallow-water regions, they stir water intensely, raising concerns about water quality degradation due to the resuspension of sediment. To address these shortcomings, developing underwater propulsion systems inspired by the propulsion methods of aquatic organisms from a biomimetic perspective and applying them to engineering is essential [2,3,4,5]. Notably, fish and other organisms exhibit remarkably high maneuverability and efficient propulsion performance. These biological characteristics can be used for technical innovation, considering the increasing demand for energy-efficient and environment-friendly propulsion systems.

Fish tails are non-rigid structures and can be flexibly deformed. This flexibility is believed to contribute to reduced fluid resistance and efficient thrust generation. Numerous studies have suggested that elastic fins are more advantageous than rigid fins in underwater propulsion research. However, the optimal stiffness of fins has been reported to vary depending on the oscillatory motion speed and corresponding swimming velocity. In practice, use of fins with different stiffnesses to achieve optimal performance is not feasible. Therefore, adjusting the stiffness dynamically during oscillation is desirable to maintain the propulsion efficiency under varying conditions. Consequently, considerable efforts have been made to integrate variable stiffness mechanisms into fins to adjust their stiffness in real time [6,7,8,9,10,11,12,13,14,15,16,17]. Many researchers have explored various methods, such as using springs with variable effective lengths and applying elastic torsion plates to dynamically alter the stiffness of the fins [18,19]. Moreover, detailed observations of the swimming behavior of aquatic organisms have revealed that during high-speed swimming, they suppress the activity of slow-twitch muscles (which are stiff and less dense, making them relatively soft overall) and efficiently utilize fast-twitch muscles (which are soft and dense, making them relatively rigid overall) [20]. This suggests that natural organisms dynamically alter the stiffness of their fins, which further emphasizes the importance of variable-stiffness fins.

However, the implementation of variable-stiffness mechanisms requires complex mechanisms, actuators, energy sources, and control systems, which present challenges in terms of robustness and miniaturization. Therefore, replacing the fins with “smart materials” that can autonomously adjust their stiffness to the optimal level is considered ideal. Previous studies have demonstrated that soft fins are advantageous for low-speed and gentle oscillatory motions, whereas stiff fins are preferable for high-speed and large oscillatory motions [18,19]. Thus, Kobayashi and Sugiyama [21] developed fins that eliminated the need for a variable-stiffness mechanism by embedding a composite fluid of shear-thickening fluid and fibers inside the fins, allowing the fins to change their stiffness according to the motion speed. The viscosity of a shear-thickening fluid is low at low shear rates and increases at high shear rates. Consequently, during slow oscillatory motion, the low viscosity of the shear-thickening fluid results in lower fin stiffness, whereas, during fast oscillatory motion, the high viscosity increases the stiffness of the fin, thereby maintaining a high propulsion efficiency across different oscillatory frequencies [22]. The incorporation of fibers can significantly enhance the local shear rate, further strengthening the velocity-dependent stiffness. In addition, it can help prevent particle sedimentation in the shear-thickening fluid, which is a key advantage of this suspension. However, as the material is a liquid, strict sealing is required, and its long-term use still faces challenges in preventing complete particle sedimentation. This indicates the need for further improvement for practical applications. Thus, few scholars focused on shear-stiffening gels as alternatives to shear-thickening fluids. Shear-stiffening gels exhibit stiffness changes according to the magnitude of the external force (strain rate), enabling velocity-dependent stiffness variations similar to those of shear-thickening fluids [23,24]. Moreover, their gel-nature eliminates the need for strict sealing, and particle sedimentation does not occur. Therefore, it is suitable for long-term use. Therefore, the use of a shear-stiffening gel could lead to the development of practical smart material fins as substitutes for shear-thickening fluids, enabling sustained optimal stiffness for applications in actual underwater propulsion systems and fish-like robots.

This research was aimed at further advancing such variable-stiffness fins for bio-inspired underwater propulsion systems and achieving complex operational tasks such as water surface lift-ups. Specifically, the envisioned applications include holding objects underwater or near the water surface without lifting them directly from above and maintaining the posture of the robot on the water surface for observation. Such functionalities can be realized by focusing on the “tail stand” movement performed by dolphins, which serves as the subject of biomimetics. In this study, we (1) leveraged biomechanical knowledge to elucidate the tail-standing mechanism of dolphins and translated it into an engineering propulsion mechanism; (2) optimized fin operation patterns, shapes, materials, and stiffness variations to achieve high thrust, even in a stationary fluid; and (3) established stabilization techniques for inverted pendulum systems using underwater fins to maintain upper-body stability and complete water surface lift-up operations. By following these steps, we attempted to enable versatile robotic operations both underwater and on a water surface. Several researchers have investigated the biomechanics for analyzing the tail stand of dolphins by calculating the thrust from vortex circulation using bubble particle image velocimetry (bubble DPIV) based on bubbles generated during the tail stand [25] and examining tail fin movements and flexibility at three tail-stand positions (low, medium, and high) [26]. Additionally, computational fluid dynamic analyses have been conducted to estimate the propulsive performance of dolphins through the analysis of the tail stand and to determine the effect of caudal fin flexibility on propulsive efficiency [27,28]. In this study, we focused on step (2), specifically “enhancing thrust in stationary fluid by modifying fin operation patterns, materials, and stiffness”. In this paper, instead of employing a simple sinusoidal oscillatory motion of the fins, we propose rapid movement of the fins and increasing their stiffness during the interval in which the thrust is positively maximized to enhance thrust generation. Conversely, during the interval in which the thrust became negative, we intentionally reduced the movement speed of the fin and stiffness to minimize the resistance. For the fin material, the aforementioned shear-stiffening gel was used. This gel enables velocity-dependent stiffness changes, allowing a single fin to become stiff during high-speed oscillations and soft during low-speed oscillations. By combining controlled oscillatory motion and velocity-dependent stiffness changes with smart materials, we attempted to improve thrust in stationary fluids and a high-thrust propulsion mechanism capable of contributing to underwater lift-up tasks.

In this study, the synergistic effects of the following two approaches were examined: (1) controlled oscillatory motion and (2) stiffness variation via smart materials, and the achievable extent of thrust improvement was achieved. These results signify the contribution of our study to the development of bioinspired robots capable of performing diverse operational tasks, such as water surface lift-ups and underwater activities.

## 2. Materials and Methods

### 2.1. Fins with Encapsulated Shear-Stiffening Gel

#### 2.1.1. Principle of Velocity-Dependent Bending Resistance in Fins

**Figure 1:**
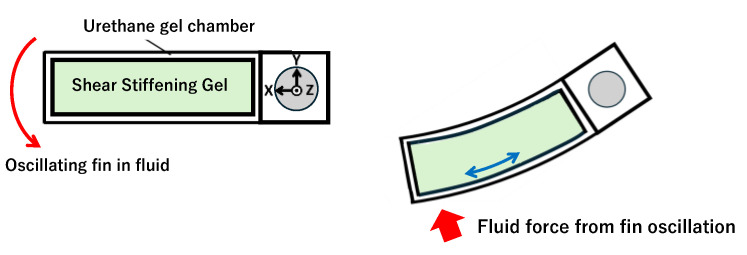
Concept of fin containing shear-stiffening gel.

In this study, a dilatant compound (DuPont, Wilmington, DE, USA, MOLYKOTE 3179) composed mainly of a silicone polymer with silica as a thickener was employed as a shear-stiffening gel. The dilatant compound can deform easily under slow shear rates while becoming stiff and exhibiting elastic properties under high shear rates. This property, known as dilatancy, gives the material its name, “dilatant compound”. For convenience, hereafter in this paper it is referred to as the “compound”. This material is used in toys such as Silly Putty, and its elasticity has been studied for applications such as impact-resistant materials [29,30,31,32,33]. As illustrated in Figure 1, we devised a rectangular fin, in which the compound was encapsulated within a bag-shaped elastic chamber. When the fin oscillates underwater, the fluid forces induce the bending of the fin, thereby generating shear rates within the encapsulated compound. In the case of a rapid oscillatory motion, the compound stiffens instantaneously, thereby increasing the overall stiffness of the fin and enhancing its apparent strength. Conversely, during the slow oscillatory motion, the compound remains in a soft state, maintaining a low fin stiffness. Thus, the stiffness of the fin changes according to its deformation speed, enabling a consistently high thrust during underwater propulsion. This velocity-dependent stiffness change was autonomously achieved by the fin in response to the shear rate experienced by the fin without the need for complex external mechanisms or actuators. Therefore, the mechanism for the variable stiffness was simplified, enabling a robust design, which is a significant advantage.

#### 2.1.2. Measurement of Viscoelasticity of Shear-Stiffening Gel

**Figure 2:**
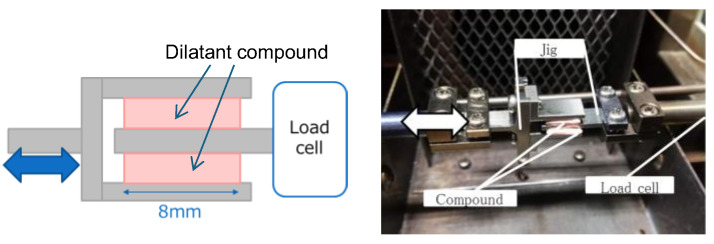
Specimens of dilatant compounds for dynamic mechanical analysis measurements.

To confirm the shear-stiffening properties of the compound (a velocity-dependent increase in stiffness), its viscoelastic properties were measured using a dynamic mechanical analysis (DMA) system (DVA-200, IT Measurement & Control Co., Ltd., Osaka, Japan). As shown in Figure 2, two compound specimens with dimensions of 6 mm × 8 mm × 2 mm were prepared and installed by clamping them to a specialized jig. The measurements were performed while varying the oscillation frequency from 0.01 to 100 Hz.

#### 2.1.3. Structure of the Fins

**Figure 3:**
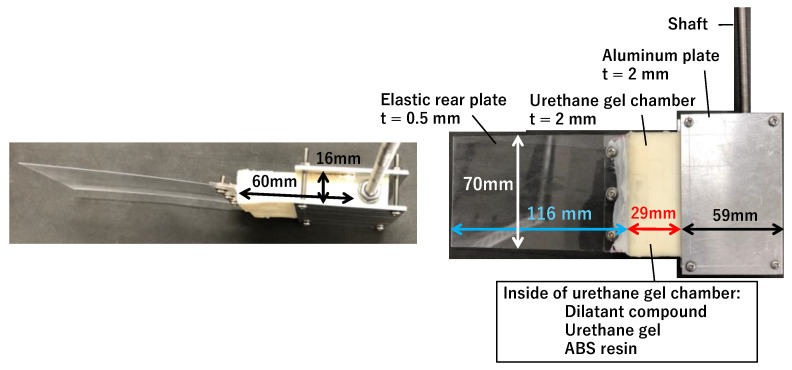
Structure of the fin.

**Figure 4:**
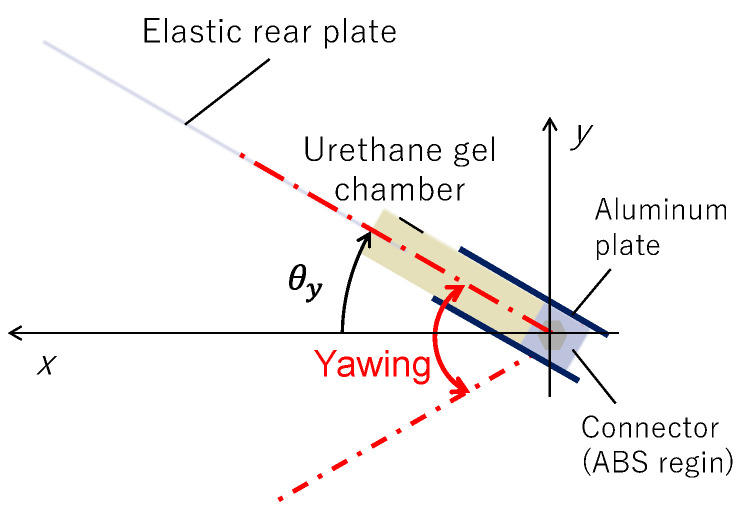
Yawing motion of fin.

As shown in Figure 3, the rectangular fins used in this study consisted of a urethane gel chamber made of urethane gel, an elastic rear plate made of PET, and a 2-mm-thick aluminum plate to secure the urethane gel chamber. The urethane gel chamber acts as a joint between the elastic rear plate and aluminum plate. The elastic rear plate had a width, length, and thickness of 70, 116, and 0.5 mm, respectively, and it was secured through a reinforcing plate connected to the urethane gel bag with bolts. The urethane gel chamber had a width, length, depth, and thickness of 70, 60, 16, and 2 mm, respectively. However, the effective length was ~29 mm when considering the area fixed by the aluminum plate. The urethane gel chamber was molded by creating a mold and core data using 3D-CAD, manufacturing the mold using a 3D printer, and then pouring a soft polyurethane-specific solvent and curing agent into the mold to form the gel. The urethane gel was the same type as that used in fins filled with a fiber-composite shear-thickening fluid in our previous studies [22] with a Shore C hardness of 40 (~1 MPa in Young’s modulus), typically used for measuring the hardness of rubber-like materials. Although urethane gel also exhibits viscoelastic properties, its G’ does not increase sharply with increasing oscillation frequency [22], and its hardening behavior in response to the shear rate can be disregarded. In this study, a fin with a slit on the shaft side of a urethane gel chamber encapsulating the compound was used. This fin is hereafter referred to as “compound joint”. For comparison, we prepared fins that behaved as rigid bodies by filling the urethane gel bag with hard ABS resin instead of the compound, referred to as “rigid joint”. In addition, a fins with a solid rectangular block of urethane gel but without a 2-mm-thick chamber shape, referred to as “urethane joint”, was fabricated. The total weight of the compound joint was ~1600 g, with no significant weight difference compared with the other fins. As the experiments involved underwater motion, the effect of the differences in the inertial forces among the fins was minimal. Initially, a design with only the urethane gel chamber was considered for the entire fin. However, to avoid an excessive weight and ensure sufficient thrust generation, the design was modified to include an elastic rear plate. This approach mimics the tail fin of a fish, where the tip undergoes passive elastic deformation and the stiffness and mechanical properties near the base are adjusted to enhance thrust. Thus, the structure was designed to achieve high thrust by appropriately controlling the material properties and behavior in the base region. Figure 4 shows the representative motion of the fins. Here, the initial investigation was focused solely on simple yawing motion, with its rotation angle defined as *θ_y_*.

#### 2.1.4. Measurement of the Bending Resistance of the Urethane Gel Chamber

**Figure 5:**
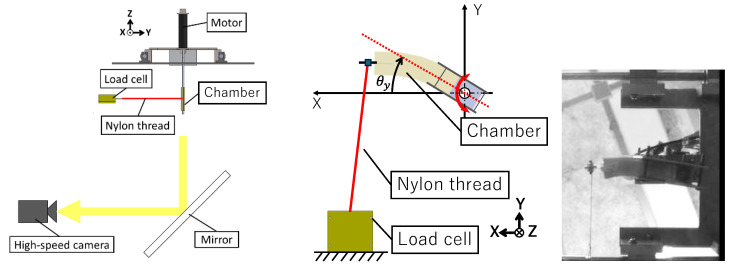
Experimental setup for measuring the bending resistance of urethane gel chamber and an example of the recorded video image (compound joint).

Figure 5 shows the experimental setup used to measure the bending resistance of the urethane gel chamber, along with an example of the recorded video image (compound joint). The measurements were conducted in air rather than underwater using a servomotor (118755, Maxon Motor, Sachseln, Switzerland) attached to the fin shaft, as shown in Figure 4. The tip of the urethane gel chamber was fixed to a load cell (T1-1000-240, AND/Orientic, Tokyo, Japan) using a nylon thread. The fin was rotated from its initial position parallel to the X–axis to a maximum rotation angle of *θy*-max = 8°. During this process, the tension applied to the thread by the displacement generated by the servomotor was measured and evaluated as the bending resistance of the urethane gel chamber. The rotation speed of the servo motor was controlled using LabVIEW 7.1 (National Instruments, Austin, TX, USA) on a PC, which sent command voltages to the servo amplifier (ADS 50/5, Maxon motor) via a DAC board (PCI6229, National Instruments). The servo motor was equipped with an encoder that allowed precise feedback control of the specified rotation speed. A sinusoidal command signal was input to rotate the urethane gel chamber, enabling a detailed investigation of the effect of mean angular velocity on the bending resistance of the fin. The bending of the chamber was videographed using a high-speed camera (HAS-220, Ditect, Tokyo, Japan), with 200 fps (resolution 640 × 480 pixels) and 8.5 mm focal length lens (Pentax, Tokyo, Japan).

### 2.2. Propulsion Mechanism Using Fins in Stationary Fluid

#### 2.2.1. Configuration, Control, and Measurement System for the Propulsion Mechanism

**Figure 6:**
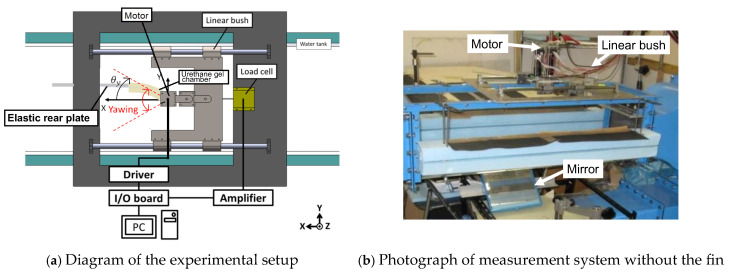
Experimental setup to measure thrust of the fin.

**Figure 7:**
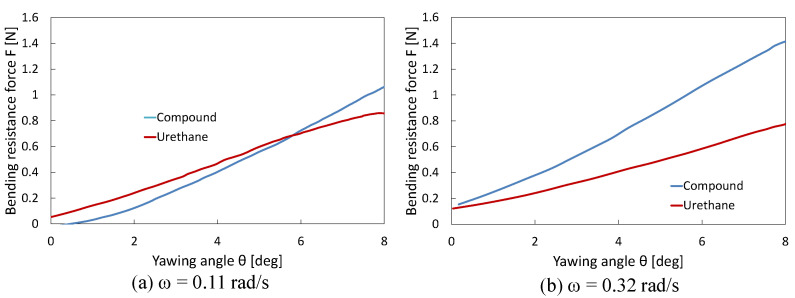
Changes in bending resistance of urethane gel chamber in compound and urethane joints at mean angular velocities (ω) of (**a**) 0.11 rad/s and (**b**) 0.32 rad/s.

As shown in Figure 6, the propulsion mechanism in the fluid consisted of a fin performing a yaw motion, a servo motor to drive and control the motion, and a load cell to measure the thrust. The position of the yawing angle *θy* is also illustrated in Figure 4. The yawing angle *θy* of the fin was measured using the encoder output of the servo motor. As in the bending resistance measurement of the urethane gel bag (Section 2.1.4), the fin shaft was directly attached to the output shaft of the servo motor to produce yaw motion. The servo motor was controlled using LabVIEW 7.1 installed on a PC, which sends command voltages to the servo amplifier via a DAC board. The servo amplifier controlled the motor based on feedback from the encoder, achieving the desired rotational speed and angle. The propulsion mechanism was mounted on a linear guide and adjusted such that the fin was positioned within the measurement channel of the water tunnel (PT-10, West Japan Fluid Engineering Laboratory, Sasebo, Japan). The water speed was zero and its temperature was set to 21 ± 1 °C. The measurement pathway in the water tunnel was 30 cm high, 30 cm wide, and 100 cm long. In this measurement channel, the fin was positioned centrally along both horizontal and longitudinal planes. A load cell was attached to the front end of the propulsion mechanism (right side of Figure 7), allowing the real-time measurement of the thrust generated by the yaw motion of the fin. The load cell was rigidly connected to the propulsion mechanism with bolts, enabling the precise detection of the forward and backward forces generated by the entire mechanism. The study was designed with underwater propulsion mechanisms for lift-up water surfaces in mind, where fins should ideally be oriented vertically. However, they are arranged horizontally here for simplicity of measurement. The yawing behavior of the fin was videographed using a high-speed camera (HAS-220, Ditect), with 200 fps (resolution 640 × 480 pixels) and 8.5-mm focal length lens (Pentax), installed below the measurement pathway of the water tunnel.

#### 2.2.2. Yawing Methods to Enhance Thrust

**Figure 8:**
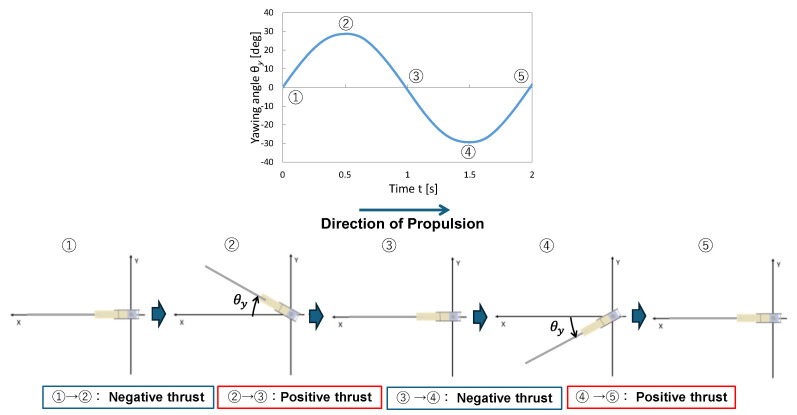
Change in yaw angle of fin and schematic representation of its position over time.

Figure 8 shows the changes in the yaw angle of the fin and a schematic representation of its position over time. The motion period was set to T = 2 s, and the deformations of the urethane gel chamber and elastic rear plate were omitted. In the figure, yawing angle *θy* = 0 corresponds to positions ①, ③, and ⑤, while the maximum angle corresponds to positions ② and ④. In intervals ① to ② and ③ to ④, the fin oscillated such that it moved in the direction of propulsion, resulting in a negative thrust during these intervals. In the intervals ② to ③ and ④ to ⑤, the fin oscillated in the opposite direction of propulsion, pushing the fluid to the opposite side of the propulsion direction, which generated positive thrust through the reactive force. By controlling the motion such that the intervals from ① to ② and ③ to ④ were “slower” while the intervals from ② to ③ and ④ to ⑤ were “faster”, negative thrust was suppressed while enhancing positive thrust. Specifically, as shown in Figure 7, the durations of ① to ② and ③ to ④ are defined as *T*_slow_, while the durations of ② to ③ and ④ to ⑤ are defined as *T*_fast_. The total duration for one cycle was set as *T* = 2*T*_slow_ + 2*T*_fast_. Here, the speed ratio is defined as

(1) $$Speed\;ratio=\frac{T_{slow}}{T_{fast}}$$

The relative yaw speed in each interval was adjusted by varying the speed ratio.

## 3. Results and Discussion

### 3.1. Viscoelasticity of Shear-Stiffening Gel

**Figure 9:**
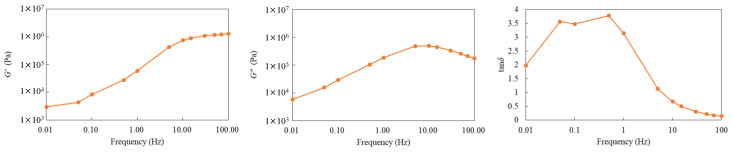
Storage modulus (G’), loss modulus (G″), and loss factor (tan δ) of the dilatant compound at 20 °C.

Figure 9 shows the measurement results of the storage modulus (*G’*), loss modulus (*G″*), and loss factor (tan δ). First, the *G’* gradually increased with increasing frequency, exhibiting more pronounced elastic behavior in the high-frequency region. Similarly, the *G″* increased with frequency, but peaked in the intermediate-frequency region. This peak indicates the frequency range in which energy dissipation is maximized, suggesting the dominance of viscous properties in this region. In contrast, tan δ exhibited relatively high values in the low-frequency range, peaked, and then decreased in the high-frequency range. This implies dominance of viscous and elastic properties in the low- and high-frequency ranges, respectively. From these results, although no abrupt stiffening (sharp increase in modulus) was observed, a gradual transition—from viscous-dominated to elastic-dominated behavior—with increasing frequency was evident. Consequently, the compound possessed sufficient shear-stiffening characteristics, with the stiffness varying with the deformation speed (shear rate). This suggests that the fins encapsulating the compound can demonstrate increased stiffness during high-speed oscillations and maintain flexibility during low-speed oscillations.

### 3.2. Bending Resistance of the Urethane Gel Chamber

**Figure 10:**
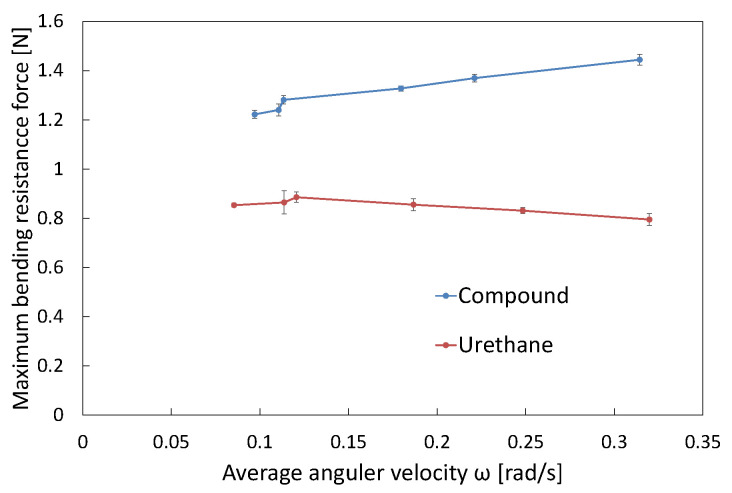
The maximum bending resistance when rotated to θ_*y*_ = 8° (*n* = 3).

Figure 7 shows the changes in bending resistance of the urethane gel chamber in the compound and urethane joints, obtained during experiments at mean angular velocities (*ω*) of 0.11 and 0.31 rad/s. According to the comparison results, the faster rotation in the latter case significantly increased the resistance of the urethane gel chamber encapsulated in the compound joint. This can be attributed to the hardening properties of the compound being further activated by a higher shear rate. Figure 10 illustrates the maximum bending resistance when rotated to *θy* = 8°, plotted against *ω*. For the urethane gel chamber in the compound joint, a clear trend was observed, where higher mean angular velocities resulted in a greater maximum bending resistance. This result supports the conclusion that the dilatancy-induced hardening behavior of the compound becomes more pronounced with an increase in the deformation speed. In contrast, the urethane gel chamber in the urethane joint (with only urethane gel) exhibited more inherent flexible properties than the compound-filled fins, with minimal changes in the maximum bending resistance as the mean angular velocity increased and even a slight decrease. This confirms that the viscoelasticity of urethane gel is relatively moderate and lacks a hardening mechanism in response to the increased shear rate. These results demonstrate that compound joints with encapsulated compounds can autonomously adjust their stiffness according to changes in deformation (rotation) speed. Moreover, comparisons with urethane joints with only urethane gel indicated that the shear-stiffening property of the compound significantly contributed to the bending resistance of the fin.

### 3.3. Yawing Angle and Angular Velocity Under Different Speedratios

**Figure 11:**
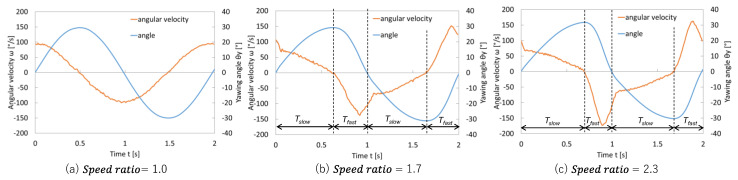
Yawing angle and angular velocity (*T* = 2.0 s, θ*ymax* = 30°).

Figure 11 shows the measured yawing angle and angular velocity. When the motion resembled a near-sinusoidal waveform, Speed ratio = 1, a slight difference was observed between the durations of the fast and slow movements. Conversely, increasing the Speed ratio value, such as 1.7 or 2.3, enabled the sections with positive thrust to be distinctly faster and those with negative thrust to be slower. This adjustment can potentially increase the overall net thrust. In the propulsion mechanism (Figure 6), angular velocity *ω* was calculated by obtaining angle data from the encoder over time and approximating it using the forward difference method. In other words, the actual angular velocity profile generated by the specified *T*_slow_ and *T*_fast_ was verified through this experimental data. Notably, when using the compound joint, the joint became relatively stiffer during the high-speed yawing in the *T*_fast_ intervals, which was expected to further amplify positive thrust. Conversely, during the *T*_slow_ intervals, the fin remained relatively soft, thereby minimizing negative thrust. This combination of velocity-dependent changes in the stiffness and high-speed ratio motion control was expected to yield synergistic effects, which was the primary objective of this study.

### 3.4. Variation in Thrust over One Yawing Cycle of the Propulsion Mechanism in Stationary Fluid

**Figure 12:**
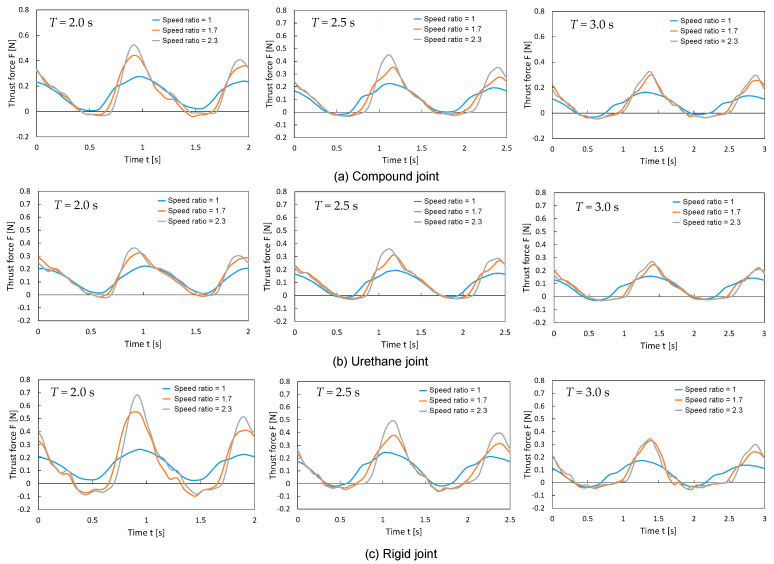
Thrust variation over one oscillation cycle for each type of fin (rigid, urethane, and compound joints) (θ*ymax* = 30°).

Figure 12 shows the thrust variation over one oscillation cycle for each fin type (rigid, urethane, and compound). The thrust data were collected after excluding the transient behaviors observed immediately after startup, focusing on the steady periodic state. For all the fins, an increase in the speed ratio resulted in a higher maximum thrust but relatively shorter durations of positive thrust generation. The characteristics of each fin are summarized below:

Rigid Joint: The positive thrust during the *T*_fast_ interval was the highest among the three types of fins. However, the negative thrust during the *T*_slow_ interval was also somewhat larger. As the urethane bag behaved essentially as a rigid body with minimal deformation, the large amplitude of the elastic rear plate likely manifested as negative thrust.

Urethane Joint: The positive thrust during the *T*_fast_ interval was the smallest among the three types of fins. Conversely, the negative thrust during the *T*_slow_ interval was minimal. The significant deformation of the urethane chamber reduced the amplitude at the connection to the elastic rear plate, likely suppressing the resistance during yaw motion.

Compound Joint: The positive thrust during the *T*_fast_ interval was intermediate, larger than the urethane joint but not as large as the rigid joint. The negative thrust during the *T*_slow_ interval was relatively small, similar to the urethane joint, but the duration of negative thrust generation was slightly longer. During high-speed yawing motions, the urethane chamber stiffened owing to shear stiffening, reduced deformation, and increased thrust. Conversely, during low-speed oscillations, the flexibility increased, but the material was inherently stiffer than that of the urethane joint, which slightly increased the overall negative thrust.

### 3.5. Deformation of the Fin in Stationary Fluid

**Figure 13:**
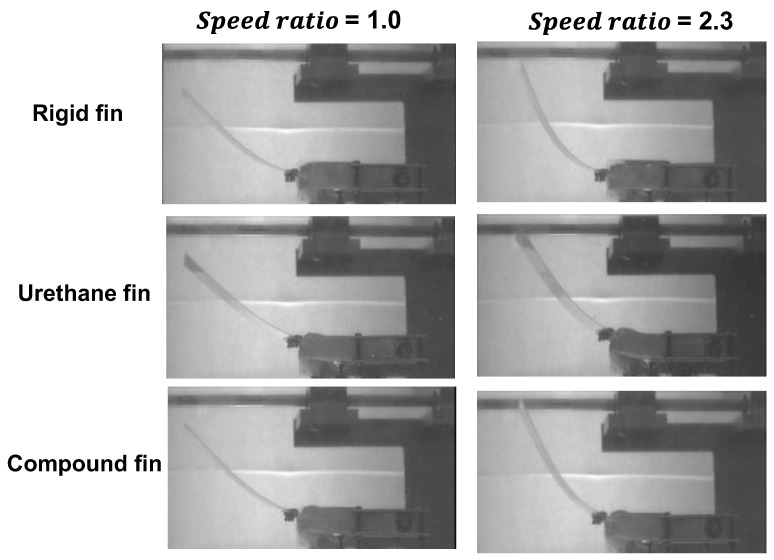
The behavior of the fin at t = 1.0 s, when the fin reaches a horizontal position (θ_*y*_ = 0°), (*T* = 2.0 s, θ*ymax* = 30°).

As part of the fin deformation during oscillatory motion, Figure 13 illustrates the behavior of the fin at t = 1 s, when the fin reaches a horizontal position (*θy* = 0°). As shown in Figure 11, an increase in the speed ratio led to a higher angular velocity at t = 1 s, which altered the inertial and hydrodynamic forces acting on the fin. The deformations of the elastic rear plate and urethane bag are summarized as follows:

Deformation of the elastic rear plate: Significant differences were observed between the fins at a speed ratio of 1. However, at a speed ratio of 2.3, the deformation was more pronounced in the following order: urethane, compounds and rigid joints. This suggests that the deformation of the urethane chamber during the high-speed Tfast interval affected the behavior of the rear plate.

Deformation of the urethane chamber: The urethane joint exhibited the most significant deformation, whereas the rigid joint showed no deformation. In the compound joint, hardening due to shear stiffening reduced the deformation, particularly during high-speed oscillations, as observed at a speed ratio of 2.3. However, the deformation did not approach “zero” as in the rigid joint but remained moderate. This probably caused the compound joint not to achieve the same peak thrust as the rigid joint, even under conditions that generated a high positive thrust. The oscillatory behaviors of the compound joint at speed ratios of 1 and 2.3 are included as Supplementary Video Materials (Videos S1 and S2).

### 3.6. Average Thrust and Thrust Variation Coefficient

**Figure 14:**
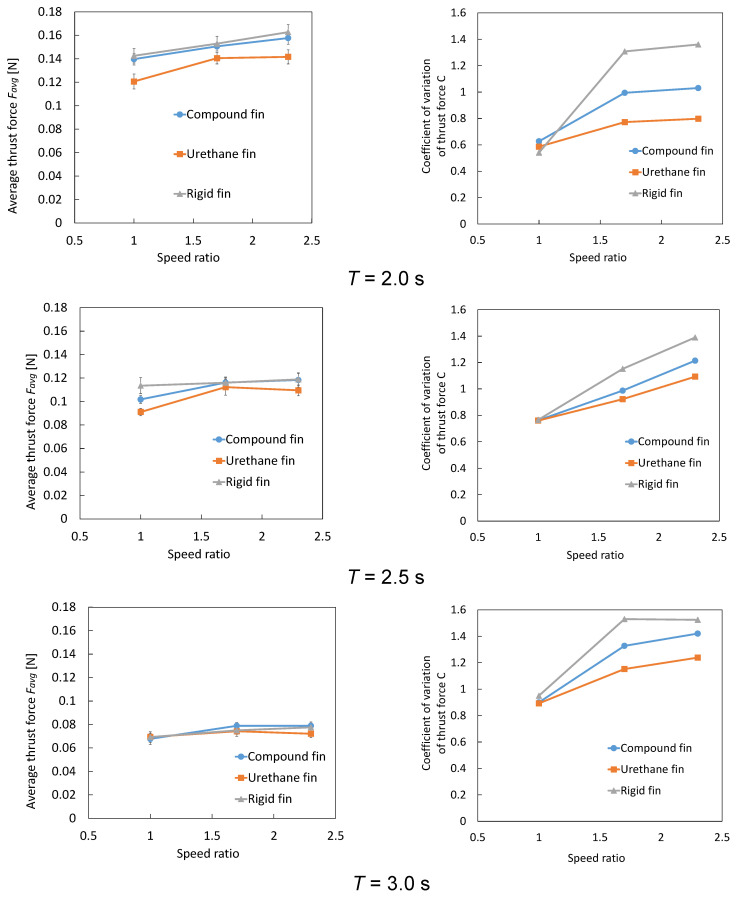
Average thrust and thrust variation coefficients for each fin type.

Figure 14 shows the average thrust and thrust variation coefficients for each fin type. Average thrust *F_ave_* and thrust variation coefficient *C* are defined as follows:

(2) $$F_{ave}=\sum_{i=1}^{N}F_{i},$$

(3) $$C=\frac{\sqrt{\frac{\sum_{i=1}^{N}{\left({F_{i}-F}_{ave}\right)}^{2}}{N}}}{F_{ave}},$$

where *F_i_* is sampled thrust data in *N* data points per oscillation cycle *T*. For the thrust variation coefficient *C*, smaller values indicate less variation in thrust within one cycle, representing more stable thrust generation.

Average Thrust: Under the cycle *T* = 3 s condition, with a low oscillation speed and relatively long cycle duration, a slight difference was observed in the average thrust among the three fin types. However, for the urethane joint, the average thrust did not increase significantly even when the speed ratio was increased. This is likely because the significant deformation of the urethane chamber makes it less effective at improving the thrust. Under the *T* = 2.5 s condition, compared to *T* = 3 s, the average thrust increased for all fins. However, the increase in the average thrust for the urethane joint was smaller than that for the other fins, and it further slowed or stagnated when the speed ratio was increased to 2.3. This is presumed to be due to the excessive deformation of the urethane chamber, which suppresses thrust improvement. In addition, the compound joint produced a lower average thrust than the rigid joint at low speed ratios. However, its thrust can improve significantly as the speed ratio increases. Under the *T* = 2 s condition, where the oscillation cycle was further shortened, the average thrust increased significantly for all fins. However, similar to the previous conditions, the urethane joint showed a slight increase in the average thrust, even when the speed ratio was increased to 2.3. The average thrust of the compound joint was slightly lower than that of the rigid joint across all speed ratios but showed consistent improvement as the speed ratio increased. Based on a simple evaluation using “average thrust alone”, the rigid joint generally performs the best. However, the compound joint benefits from shear stiffening at high-speed ratios, which effectively increases stiffness and significantly enhances average thrust.

Thrust Variation Coefficient: Under the *T* = 3 s condition, at low speed ratios, the thrust variation coefficient showed slight difference among the three fins. However, as the speed ratio increased, the values increased in the order of urethane, compound, and rigid joints. Under the *T* = 2.5 and 2 s conditions, similar to the previous results, differences in thrust variation coefficient among fins were not prominent at low speed ratios. However, at higher speed ratios, the thrust variation coefficient increased for all the fins. The compound joint demonstrated a smaller increase in the thrust variation coefficient than the rigid joint, indicating a more balanced performance.

In summary, the urethane joint, which has a slightly lower average thrust, provided a more stable thrust owing to smaller variations, whereas the rigid joint maximized the average thrust, but tended to exhibit larger fluctuations. The compound joint was not the best in terms of either average thrust or thrust variation, however, it achieved the highest overall balance. The compound joint did not exhibit the best values for either the average thrust or thrust variation coefficient. However, its average thrust was only slightly lower than that of the rigid joint, and its thrust variation coefficient was significantly lower than that of the rigid joint. Therefore, the compound joint was evaluated to be the most balanced among the three types of fins in terms of achieving both the high and stable thrust.

### 3.7. Limitations and Areas for Improvement of the Fin

This study was aimed at developing and comparing a compound joint to achieve the highest thrust across a wide range of conditions, from low to high speed ratios. Although the balance of thrust characteristics was favorable for the compound joint, its overall thrust did not exceed that of the rigid joint, and its low thrust variation surpassed that of the urethane joint. This is likely because the conditions under which the shear-stiffening (velocity-dependent hardening) function can be fully realized have not yet been adequately understood or implemented. Therefore, future studies should comprehensively explore the effects of fin scale and the associated relationship with fin stiffness, as well as the formulations, structures, and oscillation speed ranges, to maximize and optimize the shear-stiffening effect under various operational conditions. In particular, it is necessary to examine the influence of water temperature and evaluate the durability of saltwater for marine applications. In addition, because these fins used a common elastic rear plate and only varied the stiffness characteristics of the joints, the stiffness of the elastic rear plate should be varied in future studies. Furthermore, it will be possible to investigate a biomimetic design approach for fins and verify its effectiveness. Kobayashi and Sugiyama demonstrated the enhancement in the effects of a shear thickening fluid, which shares dilatant properties, by incorporating fibers [22]. We are also pursuing a similar approach and working on improving the stiffness variation of the compound by incorporating fibers [34]. However, no experiments have been conducted to incorporate compound joints into underwater propulsion mechanisms. In the future, incorporating fiber-reinforced compound joints into propulsion mechanisms and verifying their performance in underwater environments will be critical.

This study was primarily focused on evaluating the results from the perspective of thrust enhancement, with potential applications in underwater lift-up areas. However, in the development of propulsion mechanisms, propulsion efficiency and energy efficiency, which correspond not only to the stationary fluid but also to the changing swimming speed during swimming, and durability against wear over time are also critical factors that require further investigation. Moreover, to quantitatively understand the deformation behavior of the fin and surrounding fluid flow and the mechanisms for improving thrust and propulsion efficiency, flow visualization and numerical analysis techniques should be used. Furthermore, although the current rectangular design combined with an elastic rear plate enhanced thrust, tasks involving high-speed horizontal swimming or advanced maneuverability may benefit from a crescent-shaped fin similar to the tail fin of a dolphin. In the future, efforts will be focused on optimizing the fin shape to achieve greater thrust and efficiency, as well as on developing a system that measures fin deformation in real time to generate optimal motion. Additionally, it is necessary to reassess the materials and consider other materials or combinations thereof.

## 4. Conclusions

This study was aimed at using the characteristics of a shear-stiffening gel, which increases stiffness in response to deformation speed, to maintain optimal stiffness of the fin and achieve high-thrust underwater propulsion. Specifically, a “compound joint” encapsulating shear-stiffening gel was fabricated and evaluated in comparison with a “rigid joint” and a flexible “urethane joint”. Furthermore, a “speed ratio” control was implemented, designating high-speed oscillations for intervals with significant positive thrust and low-speed oscillations for intervals with negative thrust. This approach was aimed at further leveraging the velocity-dependent hardening property of the gel, enhancing the net thrust, and reducing thrust variation. The following conclusions were drawn:

(1) The compound joint exhibited an intermediate performance between that of the rigid and urethane joints, effectively reducing thrust variation while still maintaining a relatively high thrust. The key advantage of the compound joint was its ability to adjust the stiffness based on the oscillation speed, leveraging the velocity-dependent hardening of the shear-stiffening gel. This property contributed to both improved thrust efficiency and more stable propulsion, offering a unique advantage over traditional rigid or flexible fins.

(2) The rigid joint achieved the highest absolute thrust but exhibited a large thrust variation owing to its inability to adapt dynamically to oscillation conditions. The urethane joint, while minimizing negative thrust, was too flexible to generate sufficient forward propulsion at higher oscillation speeds. The compound joint provided the best balance for propulsion.

These findings suggest the possibility that the velocity-dependent stiffness function of the compound joint is effective in enhancing and stabilizing thrust in underwater propulsion mechanisms. In future, the incorporation of fibers into a shear-stiffening gel (a dilatant compound) is expected to further enhance its velocity-dependent properties. Furthermore, for practical application as an underwater propulsion mechanism, comprehensive studies are essential, not only on thrust, but also on propulsion efficiency, the relationship between deformation characteristics and the surrounding flow through flow visualization, and fin shape optimization.

## Data Availability

Data are contained within the article.

## Supplementary Materials

The following supporting information can be downloaded at: https://www.mdpi.com/article/10.3390/biomimetics10040198/s1, Supplementary Video S1. Video of yawing behavior of compound joint at speed ratios of 1 (*T* = 2.0 s, θ*ymax* = 30°). Supplementary Video S2. Video of yawing behavior of compound joint at speed ratios of 2.3 (*T* = 2.0 s, θ*ymax* = 30°).
